# Implementing Eccentric Resistance Training—Part 2: Practical Recommendations

**DOI:** 10.3390/jfmk4030055

**Published:** 2019-08-09

**Authors:** Timothy J. Suchomel, John P. Wagle, Jamie Douglas, Christopher B. Taber, Mellissa Harden, G. Gregory Haff, Michael H. Stone

**Affiliations:** 1 Department of Human Movement Sciences, Carroll University, Waukesha, WI 53186, USA; 2 Directorate of Sport, Exercise, and Physiotherapy, University of Salford, Salford, Greater Manchester M6 6PU, UK; 3 Kansas City Royals, Kansas City, MO 64129, USA; 4 High Performance Sport New Zealand, Mairangi Bay, Auckland 0632, New Zealand; 5 Department of Physical Therapy and Human Movement Science, Sacred Heart University, Fairfield, CT 06825, USA; 6 Department of Sport, Exercise, and Rehabilitation, Northumbria University, Newcastle-Upon-Tyne NE1 8ST, UK; 7 Centre for Exercise and Sports Science Research, Edith Cowan University, Joondalup, WA 6027, Australia; 8 Center of Excellence for Sport Science and Coach Education, East Tennessee State University, Johnson City, TN 37614, USA

**Keywords:** tempo training, flywheel inertial training, accentuated eccentric loading, plyometric training, hypertrophy, strength, power

## Abstract

The purpose of this review is to provide strength and conditioning practitioners with recommendations on how best to implement tempo eccentric training (TEMPO), flywheel inertial training (FIT), accentuated eccentric loading (AEL), and plyometric training (PT) into resistance training programs that seek to improve an athlete’s hypertrophy, strength, and power output. Based on the existing literature, TEMPO may be best implemented with weaker athletes to benefit positional strength and hypertrophy due to the time under tension. FIT may provide an effective hypertrophy, strength, and power stimulus for untrained and weaker individuals; however, stronger individuals may not receive the same eccentric (ECC) overload stimulus. Although AEL may be implemented throughout the training year to benefit hypertrophy, strength, and power output, this strategy is better suited for stronger individuals. When weaker and stronger individuals are exposed to PT, they are exposed to an ECC overload stimulus as a result of increases in the ECC force and ECC rate of force development. In conclusion, when choosing to utilize ECC training methods, the practitioner must integrate these methods into a holistic training program that is designed to improve the athlete’s performance capacity.

## 1. Introduction

As noted in Part 1 of this review [1], four of the most commonly prescribed eccentric (ECC) training methods include tempo ECC training (TEMPO), flywheel inertial training (FIT), accentuated eccentric loading (AEL), and plyometric training (PT). While there are many ways in which these methods can be used, there is currently a lack of evidence-based recommendations on how best to implement each method to realize the desired goals of each training phase. Previous recommendations [2,3] have mentioned several different ECC modes of training; however, many of the recommendations provided did not appear to be supported by scientific evidence, but were instead based upon anecdotal evidence. Given that many favorable acute and chronic architectural and neuromuscular adaptations can occur as a result of ECC training [4,5], it is clear that further recommendations regarding how and when to implement these methods are needed. Therefore, the purpose of this review is to provide strength and conditioning practitioners with recommendations on how best to implement TEMPO, FIT, AEL, and PT into resistance training programs that seek to improve the hypertrophy, strength, and power output characteristics of athletes. The authors acknowledge that some of the following recommendations may be theoretical; however, they are also based on the current body of scientific literature. 

## 2. Previous Eccentric Training Recommendations

Over the past 15 years, attempts have been made to provide recommendations regarding the implementation of ECC methods [2,3]. However, these recommendations have been limited in scope and appear to be based primarily on anecdotal evidence. It is not the intention of the current authors to discount anecdotal evidence, but to acknowledge that many of the elucidations regarding the considered ECC training methods have been based on this evidence, and that there is a need to update the practical training recommendations related to ECC training in order to reflect the contemporary body of scientific knowledge.

The ECC training recommendations made by Moore and Schilling [2] focused on “*augmented ECC loading*”, which is another term that has been used to describe AEL. This article discussed the use of dumbbells, elastic bands, and weight releasers to provide an overload during the ECC phase of plyometric exercises (e.g., drop jump or jump squat) in order to enhance the concentric (CON) phase of each movement. The authors recommended various barbell loads, weight releaser loads, total repetitions, and rest between repetitions for “*maximal ECC loading*”*,* “*near-maximal ECC loading*”, and “*submaximal ECC loading*”. Furthermore, the authors focused on cluster-based recommendations, specifically, exercise prescription based on performing one repetition at a time. In order to provide strength and conditioning practitioners with the best training practices, a discussion of the previous recommendations is needed. First, the minimum number of repetitions recommended was 5–10 repetitions performed with 75–85% of one repetition maximum (1RM) on the barbell, 40–55% 1RM on the weight releasers, and 45–90 s of rest in between repetitions. While 5–10 repetitions may permit the targeting of specific fitness characteristics (e.g., hypertrophy, strength, and power output) when using a cluster model, this may not be the case if a traditional resistance training (TRT) method where all of the repetitions are completed consecutively is implemented. Regarding loads, the combined barbell and weight releaser load ranged from 115–140% 1RM for the ECC portion of the movement. While the recommended barbell loads (i.e., 75–85% 1RM) may be lifted for the prescribed number of repetitions, it should be noted that these loads coincide with 10RM (75% 1RM) and 6RM (85% 1RM) values [6]; however, this is greatly individualized and may depend on the exercise performed and an individual’s level of fatigue. Thus, it is possible that the rest period between repetitions will allow these near maximal/maximal repetitions to be performed without issue; however, it is important to consider an athlete’s capacity to tolerate an additional 40–55% 1RM during the ECC phase of the movement for the same number of repetitions, given that this loading scheme may lead to training to failure. Given that the above loading recommendations are typically based on ECC/CON 1RM values, it may be important to perform a 1RM of the ECC phase of the movement to determine the loads that an individual can tolerate to ensure that the muscle force output and tension is maintained throughout the required ROM. Previous research has demonstrated that this may be accomplished by using a lowering cadence that includes light-emitting diodes during a pneumatic ECC leg press (5 s) [7] or a metronome during an ECC squat (3 s) [8]. Finally, it is important to consider the amount of rest taken within a single set of exercise. For example, if 45 s of rest was used between repetitions, an extra three minutes (e.g., 5 repetitions) to six minutes and 45 s (e.g., 10 repetitions) would be needed to complete one set of a given exercise. Thus, the length of each set prescribed may increase the overall training time, which may be limited based on the competitive season of the athlete. 

Ten years after the previous recommendations of Moore and Schilling [2], Mike et al. [3] discussed the implementation of four different ECC training techniques including the “*2/1*” (lifting a load concentrically using both limbs and return the weight eccentrically with one limb), “*two-movement*” (performing a multi-joint movement followed by the ECC action of an isolation exercise), “*slow/superslow*” (performing an ECC action using a “*superslow*” tempo and then performing the CON action with maximal intent), and “*negative/supramax*” (performing only the ECC portion of an exercise using loads greater than the 1RM of the exercise). The authors provided some information on potential ECC durations, sets and repetitions, load recommendations, rest periods, and exercise recommendations. While it appears that some of these ECC techniques may provide a unique stimulus for muscle hypertrophy and strength (e.g., tempo and negative/supermax), limited evidence supports the use of the 2/1 and two-movement techniques. Many of the exercise examples that were provided focused on single-joint movements (e.g., biceps and triceps exercises) that may not be as beneficial to an athlete’s performance when compared to multi-joint, ground-based exercises (e.g., weightlifting movements and their derivatives). In addition to the emphasis of single-joint movements, the use of the 2/1 and two-movement techniques may be questionable. The 2/1 technique recommendations call for the use of 70–80% 1RM during a 3–5 s ECC muscle action. The previous authors [3] noted that many of these movements are based on body weight and are strength-dependent; however, these recommendations appear to be solely based on anecdotal evidence. It should also be noted that while this technique may be safely used with the recommended loads when equipment that reduces anteroposterior and lateral movement (e.g., smith machine) is available, the safety of these methods may be questioned when free weight movements are utilized. When examining the two-movement technique, it is unclear what the compound (i.e., multi-joint) movement would add to the ECC stimulus that is being sought during the isolation exercise. For example, the authors suggested that a power clean and reverse curl combination with 90–110% of the athlete’s reverse curl 1RM may be used for the two-movement technique. Based on the demands of each of the movements, it is unclear how a lightly-loaded power clean performed prior to the performance of a reverse curl contributes to the overall training stimulus being sought. The negative/supermax technique with the assistance of a partner may be preferred in this situation given the focus on the ECC overload stimulus and the removal of a complex movement (e.g., power clean) that may not provide an effective training stimulus given the proposed load. 

As mentioned above, an abundance of ECC training literature has been published within the last five years that the previous authors did not have access to. As a result, the authors may have been limited in their capacity to provide evidence-based recommendations and relied instead on anecdotal evidence. Based on the limitations of the previous recommendations and the more recently published literature, it is necessary to provide strength and conditioning practitioners with the most up-to-date recommendations when prescribing ECC training methods. 

## 3. Updated Eccentric Training Recommendations

Based upon Part 1 of this two part review [1], the following recommendations can be presented for TEMPO, FIT, AEL, and PT.

### 3.1. Tempo Eccentric Training

TEMPO attempts to alter the time parameters placed on the ECC, isometric (ISO), and CON phases of the training exercise in order to elicit training responses such as hypertrophy and strength. It is postulated that by increasing the time under tension (TUT) during the ECC muscle action, greater adaptations in strength and hypertrophy can be stimulated. Furthermore, TEMPO may provide a novel stimulus for athletes and may provide benefits when returning from time off during active rest or early in the offseason during a hypertrophy/strength endurance phase. However, as discussed in Part 1 of this review, the effects of TEMPO on strength and hypertrophy are equivocal, with further research necessary to elucidate the exact training effects stimulated by this method of altering the ECC muscle action. 

One of the advantages of TEMPO is the ability to increase the total amount of TUT experienced by the muscle. As tension has been documented as the primary driver of hypertrophy [9], the use of TEMPO may be an appropriate method to develop this quality. Hypertrophy can be developed through multiple repetition ranges, but is best developed with moderate weight and moderate to large training volumes [10]. Before implementing TEMPO, practitioners should consider the potential limitations of this method. Specifically, implementing slower ECC movements may result in lower CON force, velocity, and power output [11], which may result in reduced strength adaptations. Furthermore, the deliberate slowing of the ECC muscle action may limit the contribution of the stretch-shortening cycle (SSC), which may result in a reduced transfer of training effects to sporting performance. Finally, slower ECC actions have demonstrated greater perceived effort and lactate accumulation, which may make the inclusion of TEMPO inappropriate during specific times in the annual plan [12,13,14,15]. 

The offseason may serve as an optimal time to implement TEMPO into a phase potentiation model due to its submaximal nature. During this phase of training, moderate loads may be prescribed with a high volume of training to benefit both muscle hypertrophy and work capacity [16,17,18]. Regarding the use of TEMPO for hypertrophic development, careful consideration should be given to the prescription of intensity, which will allow for a suitable amount of volume to be accumulated while including slower ECC actions. As purposefully lengthening the ECC phase may result in a lower number of achieved repetitions compared to a self-selected pace, the loading intensity may need to be lowered to complete the prescribed set and repetition scheme [19,20]. Additionally, attention should be paid to the response to this loading, which may result in higher acute fatigue and the athlete perceived exertion during the session [12,13,14]. It should be noted that although the number of repetitions may decrease with a longer ECC phase, the TUT increases [21]. Thus, practitioners must decide if the number of repetitions performed or the athlete’s total TUT is of greater importance based on the desired training goals.

TEMPO may be appropriate for athletes of all levels as long as it is administered at the correct time points in a training plan. Due to submaximal loading conditions inherent to TEMPO, it may not be appropriate during training phases that target maximal strength, maximal power output, or high-speed training. When the ECC phase is extended, it may limit the power output in the subsequent CON phase, limiting its transference during times of focused power development [19,20,22,23,24,25]. Table 1 shows a sample offseason plan with the addition of TEMPO on the primary exercises for that training microcycle. 

### 3.2. Flywheel Inertial Training

Several meta-analyses have examined the effectiveness of FIT in improving muscle hypertrophy, strength, and power output [26,27,28,29]; however, very little practical information regarding the sets, repetitions, intensities, or frequencies that can be used to optimize training has been provided. Tesch et al. [30] summarized the findings of a number of FIT studies and provided some practical guidelines for YoYo™ exercise training. Specifically, the authors recommended that FIT should be performed with four sets of seven repetitions with 90–180 s of rest between sets, no more than twice per week with 48 h of recovery between sessions. While this set and repetition scheme may vary from TRT, the majority of FIT studies have used this protocol [31,32,33,34,35,36,37,38,39,40].

Previous studies have investigated the effects of FIT performed using four sets of seven repetitions on muscle size (e.g., cross-sectional area, volume, mass) [31,32,33,34,35,36,37,38,39,40,41]. While these studies have displayed positive increases in muscle size, the participants trained using isolated, single-joint movements (e.g., knee extension) or small muscle group exercises (e.g., shoulder abduction). As a result, the conclusions drawn within the meta-analyses may be biased, given that 77.8% [26,27], 70.0% [29], and 57.1% [28] of the studies included for muscle size were based on these movements. While these exercises may be beneficial for body building and rehabilitation settings, they are not commonly prescribed when training team sport athletes. Thus, readers should be cautious when attempting to apply these findings to multi-joint exercises.

Specific to force development (i.e., strength), previous research has suggested using higher inertial loads when using FIT devices [30,42,43,44]. However, another study indicated that moderately active participants experienced no additional ECC stimulus with inertial loads beyond 0.0375 kg∙m^2^ [43]. Similar to muscle size adaptations, a number of FIT studies have displayed improvements in strength (e.g., muscle force, maximal voluntary contraction, 1RM, torque, etc.) using a set and repetition scheme similar to the discussed four sets of seven repetitions protocol [31,32,33,34,35,36,38,39,40,44,45]. Given the multi-joint nature of sporting movements (e.g., running, jumping, change of direction, etc.), strength and conditioning practitioners are encouraged to prescribe these types of movements if FIT is used as a training method. With regard to the ECC overload stimulus that contributes to strength adaptations, it has been recommended that individuals should gently resist the inertial force during the first third of the ECC action and then apply maximal force at the end range of motion [46]. 

In contrast to the strength recommendations above, recent studies have suggested that lower inertial resistances should be used to enhance power output [30,44]. Several studies have reported improvements in power output or explosive performance (e.g., countermovement jump (CMJ), squat jump, running speed, etc.) [31,32,33,34,36,37,44,47,48,49,50,51,52]. While many of these studies used protocols similar to those discussed above (i.e., four sets of 7–8 repetitions), other studies reported positive results using three sets of 15–20 s [48,50,51]. A recent meta-analysis concluded that well-trained individuals displayed greater power output adaptations when compared to untrained and moderately-trained individuals following various FIT protocols [29]. It should be noted that the authors defined “well-trained” as “sport-participating individuals, elite athletes, and individuals with self-reported high activity level.” Within their analysis, several improvements in power output stood out including increases of 32.6% in five weeks [48], 30.0% in six weeks [37], 38.2% in seven weeks [44], and 59.2% in 24 weeks [49]. These are very large increases in power output over short periods of time and should be interpreted with caution. In addition, it should be noted that all of the highlighted studies measured power output during the FIT training protocol [37,44,48,49]. Thus, it is possible that the novel training stimulus led to greater performance improvements within the task they were performing. Interestingly, two of the previous studies, despite improving power output during the performance task, displayed only a trivial-small increase in CMJ performance [37,49], which is a test that is more commonly used to monitor power output. Therefore, the use of FIT for improvements in power output may be effective in weaker, less-trained individuals; however, limited evidence suggests that the same benefits can be achieved with stronger, well-trained individuals.

Several recent studies have examined the potentiation effects of FIT on various performance variables [53,54,55,56]. The results of these studies indicated that FIT may improve swimming start force variables [55,56], squat jump performance (e.g., height, velocity, and power output) [54], and CMJ performance (e.g., height, peak power output, and impulse) [53]. From a FIT prescription standpoint, the potentiation studies used either one set of 3–4 repetitions using a swim start lunge position (one additional repetition performed to get the FIT up to speed) [55,56] or three sets of six half-squat repetitions [53,54]. Interestingly, only one of the previous studies provided inertial load information within their methodology [53], making it difficult to recommend a specific protocol for stimulating potentiation effects. Based on the extant literature, low volumes of FIT may allow individuals to realize potentiation effects; however, further research is needed that compares different FIT and TRT protocols as well as individuals with different training backgrounds (e.g., strength levels, experience with FIT device, sport participation, etc.).

**Figure 1:**
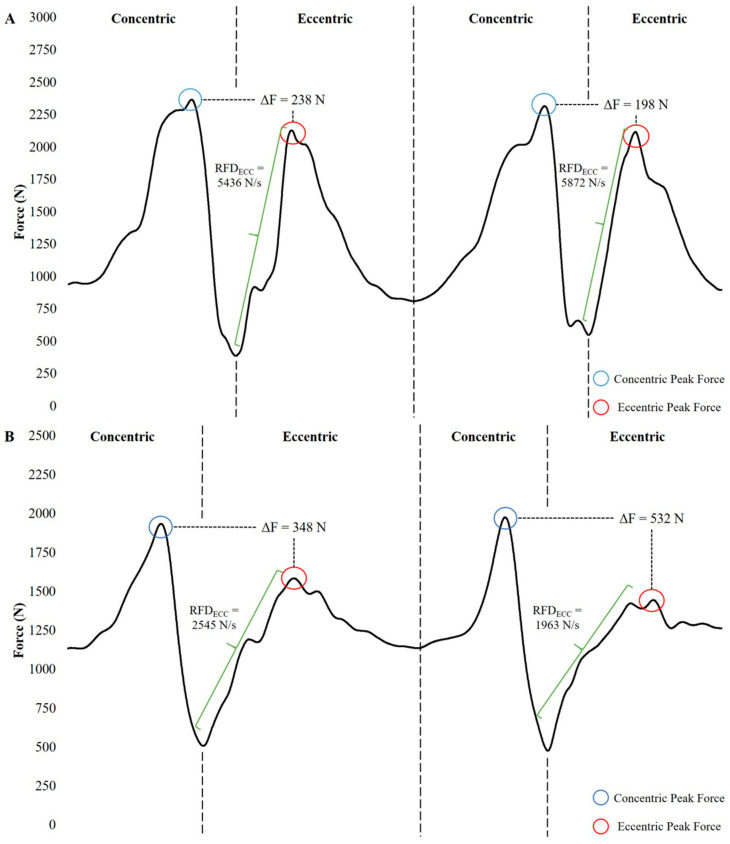
Force–time curves of flywheel squats performed with a fast-concentric action and slow-eccentric action by a weaker, heavier male (**A**) and a stronger, lighter male (**B**).

While it appears that four sets of seven repetitions may be an effective FIT protocol for hypertrophy, strength, and power output, limited information exists on whether this is optimal for all populations. To the authors’ knowledge, only one study has provided information regarding the relative strength of their participants [44], despite several others providing measures of absolute strength [31,34,41,48,57]. From a strength perspective, stronger athletes require a greater overload stimulus than weaker athletes due to their capacity to tolerate heavier loads. Figure 1 displays the force–time curves of two healthy males with largely different relative strength levels (2.4× vs. 1.5× body weight squat) performing flywheel squats with the same inertial load. Although this is just one example, this comparison demonstrates that a stronger individual may not receive the same ECC overload stimulus as that of a weaker individual, likely due to the fact that the stronger individual is able to adapt a more compliant strategy that allows them to slow down the ECC load and control the movement to a greater extent. Regardless of training experience, it is recommended that individuals perform the CON phase of the movement as quickly as possible if the goal is to receive a larger ECC overload stimulus [1]. 

Based on the available evidence, it is suggested that FIT may best serve untrained individuals, weaker athletes, and those who are going through rehabilitation and return to play protocols. While this conclusion may be in contrast to a recent meta-analysis [29], readers are encouraged to carefully read the individual studies cited within each meta-analysis to understand that the vast majority of the participants would, at best, be classified as moderately-trained based on their resistance training experience. Previous research suggests that early prescription of ECC training may benefit hypertrophy and strength of muscles surround the knee following anterior cruciate ligament (ACL) reconstruction [58,59,60]. Furthermore, the benefits from FIT during the rehabilitation of injuries has been reported in the scientific literature [49,61,62]. From a rehabilitation perspective, the way a FIT device can serve an athlete is twofold. First, part of a return to play protocol will likely include force absorption exercises, whether they are performed bilaterally or unilaterally. The benefit of FIT in this regard, is that individuals may be able to use their movement velocity to gauge intensity while the resistance experienced is variable. Second, a FIT device may allow an individual to re-learn how to effectively transition from an ECC action to a CON action, which is performed frequently during sporting situations. Thus, FIT may work as a motor learning device when used in this manner. 

### 3.3. Accentuated Eccentric Loading

When programming AEL, strength and conditioning practitioners must follow three basic tenets: (1) the ECC load is in excess, in absolute terms, of the CON load, (2) the exercise selected involves a coupled ECC and CON action, and (3) the mode of applying the overload imparts minimal interruption to the natural technique of the exercise selected. As mentioned in Part 1 of this review [1], AEL has demonstrated the ability to improve strength and power production when prescribed chronically, which is likely due to either changes in the nervous system [63], more favorable size and phenotypic changes to fast muscle isoforms [64], or an enhanced anabolic environment [65] compared to TRT. AEL has also been used to exploit acute potentiation effects due to its mechanistic ability to increase ECC rate of force development (RFD_ECC_), thus creating a greater rate and magnitude of stretch to the musculotendinous unit [66] and an augmented myotatic reflex. Overall, AEL appears to be a versatile programming tactic with demonstrated efficacy pertaining to several concentrated loading stimuli within a sequenced training process. Interestingly, previous literature has indicated that stronger individuals may benefit more from using lighter relative loads (e.g., 105–110% 1RM) during the ECC phase of an exercise, while weaker individuals may benefit more from using heavier loads (e.g., 120–130%) [67]. While this recommendation may provide practitioners with a starting point, further research is needed in this area.

During the early stages of a phase potentiation model aimed at the development of power output, the strength and conditioning coach may use programming tactics aimed at enhancing work capacity and muscle size or architecture changes. In this regard, larger magnitudes of ECC overload with AEL may apply a higher magnitude of mechanical tension to the stressed musculature, the primary driver of muscle hypertrophy [9]. As higher magnitudes of ECC work have been observed even when the ECC overload was only applied to the first repetition within a straight set, [68], the set structure may not need to be altered in order to produce this potentially favorable tension profile. While Douglas and colleagues [69] recently demonstrated that AEL improved muscle strength independent of size changes, Shoenfeld and Grgic [70] recently suggested that conservative usage of ECC overload may be enough to augment muscle hypertrophy. Taken together, the maintenance of the set structure is likely to be most beneficial for work capacity adaptations, while supramaximal AEL may be the most beneficial for inducing muscle size changes. Therefore, when programming AEL in the early stages of the training process, supramaximal loading of the ECC phase during the first repetition only appears to be the logical recommendation of a programming strategy. It is worth noting that specialized equipment does exist that may allow for the maintenance of set structure while programming AEL [7]; however, these devices are not commonplace in most strength and conditioning settings. 

Though supramaximal AEL has a place in a maximal strength phase of training, the results of previous investigations are inconclusive [64,71,72]. Supramaximal AEL appears to be most advantageous for strength adaptations when the relative difference in percent of 1RM between the ECC overload and the CON load is larger. Though a variety of supramaximal overloads (e.g., 100–120% 1RM) have demonstrated efficacy, this is primarily the case when the difference between the ECC and CON loads is greater than 30% 1RM [64,65,73,74,75,76,77]. Though the response to varying relative differences has not been directly explored, the practitioner may pool the information from several investigations and prescribe loading with the 30% rule of thumb as a guide. As is the case with traditional training theory, strength and conditioning practitioners should also be conservative with total volume when aiming to develop maximal strength, as improper fatigue management may be detrimental to the desired outcomes of the training and possibly the technical execution of the prescribed lift. Additionally, most training processes are multi-factorial in nature and coexist alongside a technical and tactical development process. When AEL has been programmed alongside a multi-factorial process, it has diminished sprint ability and strength performance, most likely due to the concurrent nature of the training suppressing potentially favorable effects of AEL [69]. Therefore, strength and conditioning practitioners should be cognizant of the overall volume and total load relative to prior phases in the prescription of AEL with the desired outcome of maximal strength changes. However, as noted above, the strength level of the individual may dictate the load prescribed during the ECC phase of an exercise [67].

AEL also has reasonable applications in the late stages of a periodized plan for strength–power athletes. As previously mentioned, the ECC overload may increase RFD_ECC_ and provide a mechanistically favorable situation for the athlete to experience potentiation of the CON phase. By training at the highest acute power outputs and movement rates, the athlete can expect to experience greater development in power production in the long-term. In doing so, conservative ECC overload strategies have been explored, primarily applied to ballistic actions like throws and plyometrics. Most investigations have used prescriptions based off athlete body mass, ranging from 10–30%. Such investigations have observed enhancements in force production magnitude and rate, take-off velocity, peak power production, and jump height [78,79]. Therefore, when in the late stages of a periodized training plan, the optimal realization of power production potential is most likely using these conservative loading strategies.

### 3.4. Plyometric Training

As identified in Part 1 of this review [1], plyometric exercises are commonly used to optimize SSC function. Research has highlighted that better utilization of the ECC phase resulting from training adaptations in ECC phase force and velocity characteristics can result in improved CON performance [80]. Consequently, it is important for strength and conditioning practitioners to consider appropriate pathways for facilitating the development of their athlete’s maximal strength and expression of strength under different ECC conditions. The following will discuss the use of plyometric exercises and variants of plyometric exercises to offer an ECC training stimulus.

**Figure 2:**
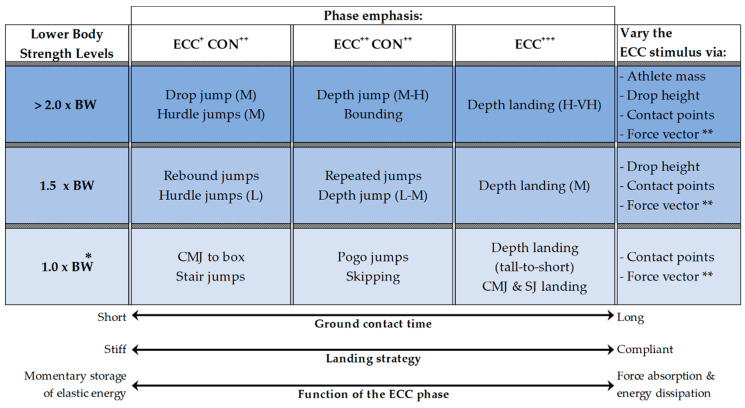
Characteristics and progressions of plyometric exercise to emphasize eccentric loading for athletes of varying strength level. ECC = eccentric; CON = concentric; + = low emphasis; ++ = moderate emphasis; +++ = high emphasis; L = low height; M = moderate height; H = high height; VH = very high height; * = overall priority on correct skill execution and landing mechanics, ** = not applicable to exercises requiring landing only (ECC^+++^ column)**.**

**Figure 3:**
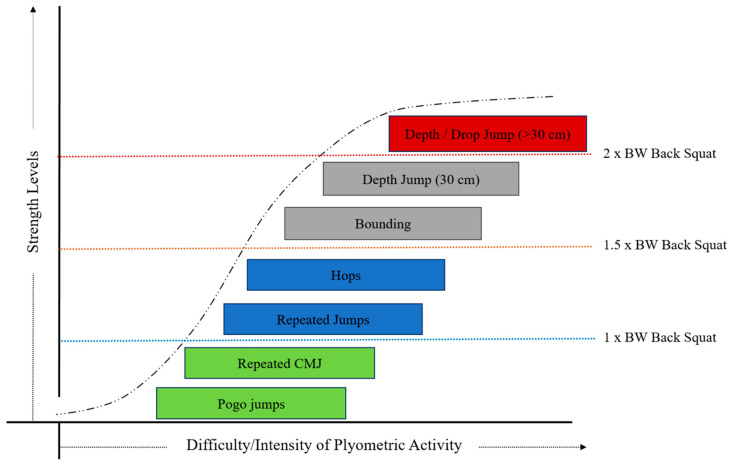
Theoretical relationship between strength and the optimization of plyometric exercise performance. Adapted from Suchomel et al. [93], Buchner et al. [99], and Haff [100] BW = body weight.

ECC force production can be emphasized using of a variety of plyometric exercises (e.g., jumping, hopping, and bounding). The degree of emphasis placed on the ECC phase can be manipulated such that it can be less than (e.g., box jump), equal to (e.g., repeated jumps), and greater than (e.g., drop jump) the emphasis placed on the CON phase. Moreover, the ECC phase can also be performed in isolation (e.g., depth landing). Hence, plyometric exercises can offer exercise intensities ranging from submaximal to supramaximal. Based upon information from numerous studies that have used ground reaction forces, RFD_ECC_, GCT, impulse, and integrated electromyography to quantify the intensity of a variety of plyometric exercises [81,82,83,84,85,86,87,88,89,90,91], the progression of plyometric exercises can be aligned with an athlete’s relative back squat strength [92,93]. Figure 2 and Figure 3 present a theoretical model of how ECC exercise intensity may be progressed for athletes within the different categorization of lower body strength levels.

Traditional recommendations for implementing PT are based on the number of ground contacts; 80–100 for beginner, 100–120 for intermediate, and 120–140 for advanced athletes [94]. A meta-analysis comprising of 56 studies clearly documents that researchers have implemented a wide variety of protocols comprising various degrees of exercise intensity levels and training volume [95]. Based upon these data, there appears to be a dose–response trend for optimizing plyometric-induced gains in vertical jump performance that appear to result from programs greater than 10 weeks in duration and are comprised of more than 20 sessions in total, with each session containing more than 50 jumps of a combination of different types of plyometric exercises (squat, countermovement, and drop jump) performed under high intensity conditions, which appeared to translate to improvements in performance. Despite the above recommendations, strength and conditioning practitioners should always be aware of the neuromuscular fatigue that may result from different volumes of PT [96] as increasing training frequency to attain greater training volume per week may not always translate to greater improvements in performance [95,97,98].

The ECC component offered by plyometric exercises that use coupled ECC-CON actions tend to adopt a stiffer strategy and short GCTs, which can result in greater tendinous lengthening and less fascicle lengthening [101,102]. Hence, the displacement of muscle fibers during SSC movements is minimal, whereby the muscle operates closer to the optimal length and on the plateau region of the length–tension relationship. Therefore, if PT is applied with the intention of performing true ECC muscle actions, then a more compliant strategy promoting longer muscle lengths may be more suitable, which could be offered by depth landing exercises. Importantly, isolating the ECC phase of plyometric exercise offers the opportunity to utilize the greater force producing capacity innate to ECC muscle actions. This approach enables the application of an overload stimulus to prompt very high muscle tension and force output, which has the potential to be greater than that offered by exercises that are limited by CON strength. Therefore, a supramaximal or overload stimulus may be applied when drop heights create a scenario where the athlete cannot efficiently transition from the ECC to CON phase. 

While ECC exercise is an important training tool, its high intensity application may be more appropriate for stronger athletes that have developed a significant ‘strength reserve’ (e.g., >2.0× body mass lower body strength) (Figure 3) [93] and require novel and potent stimuli to prompt further increases in maximal strength [93,103]. Optimal training recommendations are likely to differ from those mentioned earlier, given the high intensity nature of these exercises. However, there appears to be a paucity in recommendations for this type of exercise. Evidence suggests that the exact prescription of high intensity ECC exercise is likely to vary between athletes due to individual differences in relative strength and physiological tolerance to ECC exercise [104,105]. Instead, adopting an individualized approach to prescription is recommended. For weaker athletes, PT can be used to focus on landing mechanics under much lower (submaximal) intensity conditions until they have further developed their overall strength levels. That said, performing ECC-focused movements offer the opportunity to optimize landing mechanics and provide a means for weaker athletes to develop ECC strength [94]. In this scenario, given the lower intensity of this approach, optimal exercise prescription is likely to better reflect the guidelines mentioned previously.

Finally, like other forms of resistance training, it is suggested that PT should be implemented in a periodized manner to prescribe the appropriate volumes and intensities needed to realize the desired training adaptations and minimize fatigue [96,106]. Overall, PT appears to be a versatile method that has the potential to target ECC qualities across the force–velocity spectrum. The theoretical model presented in this section displays how ECC exercise intensity could be progressed using different applications of PT that are modifiable to suit the strength level of the athlete (Figure 2 and Figure 3). PT offers a means to apply an overload stimulus during the ECC phase using non-specialized equipment, which is innate to the other methods of ECC training addressed in this review.

## 4. Additional Programming Considerations 

While the ECC training recommendations mentioned above provide some insight on how to implement TEMPO, FIT, AEL, and PT, several other considerations must be taken into account. Specifically, strength and conditioning practitioners must consider an athlete’s training experience, relative strength, the adaptations that are being sought during specific training phases, and the ability to integrate ECC training into a holistic resistance training program that will benefit an athlete’s overall performance.

### 4.1. Training Experience

The resistance training programs prescribed for athletes are often dependent on their technique competency. As a result, novice (weaker) athletes should not be loaded too quickly until they develop a motor program and technique that will allow them to control a given load during the ECC and CON phases of a lift. While these individuals will be progressively loaded, it is important for the strength and conditioning practitioner to program in a logical manner that will not make the movement too variable (e.g., weight released at certain periods). Therefore, if some of the ECC training methods discussed within this review are prescribed too early in an athlete’s development, it is possible that negative adaptations may occur. Furthermore, if introduced too early, these methods may then have reduced novelty as a training stimulus later, which may adversely influence their effectiveness. Thus, from an ECC standpoint, weaker athletes may benefit from incorporating TEMPO and FIT to develop positional strength as well as learn how to accept/absorb an ECC load. 

While the primary focus of weaker, less experienced individuals may be to gain and improve their absolute and relative strength, stronger, more experienced individuals may require a novel training stimulus to continue to improve their performance capacity [92]. Previous studies have indicated that this may require a shift in their training focus to more power/ballistic-type training [107,108,109]. From an ECC training standpoint, this may require a greater overload stimulus in the form of greater ECC force and/or RFD_ECC_. As discussed previously, stronger athletes may not receive the same overload stimulus as weaker individuals during TEMPO and FIT ECC methods. However, the exception to this may be the relative intensity and TUT combination that may be used during TEMPO when it is used as a hypertrophy stimulus. In contrast, stronger individuals may benefit more from AEL and high intensity PT than their weaker counterparts. For example, previous research suggests that stronger individuals may benefit more from advanced methods of training such as PT [110,111]. This is likely due to their ability to tolerate greater ECC force and RFD_ECC_. Table 2 provides recommendations of which ECC training methods may be the most appropriate for athletes with different training ages based on relative strength. 

### 4.2. Phase Specificity

An important consideration when programming various ECC training methods is the time course of the desired adaptation(s). Like any other training method, it is important to plan training ahead of time to maximize performance adaptations during the competitive season (e.g., team sports) as well as during the most important competitions within a season (e.g., track and field). It should be noted that certain performance adaptations may require longer periods of training time to maximize their potential (e.g., RFD and power output). Moreover, the physiological adaptations are exploitative in nature, emphasizing the role of the proper sequence of training contents. Previous literature has indicated that increasing work capacity may increase an individual’s potential to gain strength and in turn, these improvements in strength may enhance an individual’s potential to improve their power output [16,17,18,108]. While this concept, termed phase potentiation, is primarily discussed in terms of TRT, the same training theory can be applied to ECC training methods. Specifically, ECC training methods may lead to phase specific adaptations that may benefit an athlete’s overall performance.

**Figure 4:**
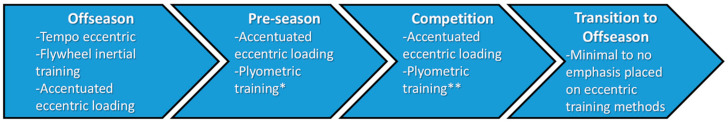
Recommended eccentric training methods based on the different phases of the training year. * Moderate-high intensity plyometric exercises may be prescribed to enhance speed-strength adaptations; ** High intensity plyometric exercises may provide a unique overload stimulus that may enhance eccentric rate of force development characteristics.

Figure 4 displays the theoretical progression of ECC training methods throughout the training year. Often, the initial goals of a resistance training program are to improve an athlete’s work capacity and increase the muscle cross-sectional area [107,109]. This type of work is often completed in the offseason phase of training due to the high volume of work and fatigue that accumulates. Based on the TUT, the requirement to overcome an ECC load, and ability to overload the ECC phase, TEMPO, FIT, and AEL may have the greatest potential to improve these characteristics, respectively. Following this phase, athletes then shift their focus to improving force production (i.e., strength) and early RFD characteristics. From an ECC training perspective, a combination of AEL and PT may provide the greatest potential to enhance these characteristics. As discussed above, and in Part 1 of this review [1], AEL may have the greatest potential to improve strength characteristics due to its ability to overload the ECC action of a movement. This in turn may also enhance RFD_ECC_. In contrast, PT may be limited in its capacity to provide a force overload stimulus; however, by training the ability to transition from an ECC to CON action, an athlete can enhance both RFD_ECC_ and RFD_CON_ [80]. This type of training would often be reserved for the late offseason and early preseason phases. As mentioned above, power output may be underpinned by both work capacity and muscular strength. However, another factor that may benefit this characteristic is the shortening velocity of the muscle fibers. Muscle architectural adaptations that may benefit shortening velocity include a decreased pennation angle and an increase in fascicle length. Given that power development is most often sought during the late pre-season and competition phases of the training year, specific ECC training methods may provide an optimal training stimulus during these times. During the pre-season phase, AEL may programmed using the back squat exercise; however, during the competition phase, power-oriented AEL [112] and specific high velocity plyometric exercises may further develop power output. While the previous sequenced progression may provide a framework for power development, strength and conditioning practitioners should understand the training residuals of each adaptation following the cessation of training [113] as well as the fact that not all fitness qualities decay at the same rate [114].

### 4.3. Integration with Other Training Methods

While the current review provides practical recommendations for several ECC training methods, the authors acknowledge that other training tools such as weightlifting movements and their derivatives [115,116,117,118], loaded jumping exercises [119], ECC cycling [120,121], change of direction drills [8], and various sprinting tasks may provide an ECC overload stimulus; however, practical recommendations of these methods were not included in this review due to either insufficient evidence, or the secondary nature of the training stimulus. 

Despite the training potential of ECC training methods, it is important for strength and conditioning practitioners to remember that ECC training should not, in most cases, be exclusively prescribed for an athlete at any given time throughout the training year. Instead of replacing all other methods of training, the discussed ECC training methods should be integrated into a holistic resistance training program that also features TRT (i.e., ECC/CON), ballistic exercises, and other training methods. While the training experience and/or strength levels of athletes may dictate what methods of training may be prescribed throughout the training year [92], strength and conditioning practitioners should be aware of the residual training effects that result from different methods of training and should plan which methods should be emphasized during different phases of training. This knowledge should then be used to periodize ECC training throughout the competitive year for each athlete, but also across their entire competitive career.

## 5. Summary

While ECC training prescriptions have been provided in the past, a lack of scientific literature prevented the previous authors from providing evidence-based recommendations. TEMPO may provide an effective stimulus for weaker, less experienced individuals in the form of positional strength. However, TEMPO may benefit hypertrophy characteristics of both weaker and stronger individuals by increasing the TUT. It should be noted however, that due to an increase in repetition duration, TEMPO should be avoided during a power phase. In contrast to TEMPO, FIT may provide an effective training stimulus for hypertrophy, strength, and power output. However, it is important to note that stronger individuals may not receive the same ECC overload stimulus due to their ability to tolerate and control higher ECC forces and thus, FIT may be a more effective training stimulus for weaker individuals who have less resistance training experience. AEL has the potential to be an effective training stimulus throughout the entire training year; however, this method may be better suited for stronger individuals who have the capacity to handle high ECC forces and RFD_ECC_. From a loading standpoint, some literature suggests using lighter relative loads during the ECC phase of exercise for stronger individuals and heavier loads for weaker individuals. It is also important to note that an AEL stimulus may vary based on the equipment that is used (e.g., back squat with weight releasers vs. drop jumps with dumbbells) and thus, may be used as part of a multi-faceted approach to develop specific fitness characteristics. Finally, PT has the potential to provide an effective overload stimulus in the form of high ECC forces and RFD_ECC_. While PT can be effectively implemented with both weaker and stronger individuals, is important to note that stronger individuals possess a greater capacity to tolerate ECC loading and may thus, be capable of withstanding greater volumes of PT, higher intensity plyometric exercises, and more frequent prescription. However, like other forms of resistance training, PT should be properly progressed and implemented in a periodized fashion to meet the training needs of each particular athlete.

Additional training concerns must be considered when implementing ECC resistance training. Specifically, an athlete’s previous training experience (e.g., strength levels, exercise competency, recent training phases, etc.), the goals of specific training phases, and the ability to integrate one or multiple ECC training methods with other training methods should be considered. An athlete’s relative strength may dictate which ECC training method may be the most appropriate for them. Based on the existing literature, it appears that TEMPO, FIT, and low-moderate PT may be the most appropriate methods for weaker individuals. In contrast, stronger individuals may benefit more from AEL and moderate-high intensity PT. Regarding phase specificity, it is important to implement ECC training methods that may most effectively develop the desired fitness characteristics at specific times during the training year. For example, TEMPO and FIT may better serve an individual during moderate-high volumes of training in order to develop work capacity. In contrast, AEL and PT may be best implemented during lower volumes of training in order to effectively develop strength–power characteristics. Finally, it is important that practitioners consider the recommendations made within this review as part of a holistic resistance program for the athlete. ECC training methods should not replace all other methods of training, but should complement what is already being prescribed.

## Figures and Tables

**Table 1:** Sample off-season training plan with the inclusion of tempo training to improve work capacity and cross-sectional area.

Monday		Wednesday		Friday	
1. Back squat	3 x 8 5/0/1	1. Mid-thigh pull	3 x 8 x/0/x	1. Back squat	3 x 8 5/0/1
2. Bench press	3 x 8 5/0/1	2. Deadlift	3 x 8 5/0/1	2. Bench press	3 x 8 5/0/1
3. Split squat	3 x 8 x/0/x	3. Bent over row	3 x 8 5/0/1	3. Split squat	3 x 8 x/0/x
4. Military press	3 x 8 x/0/x	4. Pull-up	3 x 8 x/0/x	4. Military press	3 x 8 x/0/x

Note: Exercise prescription is displayed as sets x reps and the tempo prescription is displayed as eccentric/isometric/concentric in seconds. x indicates that the eccentric and concentric durations may be variable.

**Table 2:** Suggested eccentric training methods for beginner, intermediate, and advanced athletes.

	Beginner (<1.0× body mass squat)	Intermediate (1.5× body mass squat)	Advanced (≥2× body mass squat)
Suggested Eccentric Training Method(s)	Tempo Eccentric Training Flywheel Inertial Training Plyometric Training *	Flywheel Inertial Training Submaximal Accentuated Eccentric Loading Plyometric Training **	Maximal to Supramaximal Accentuated Eccentric Loading Plyometric Training ***

* Low-moderate intensity plyometric exercises are recommended as the primary focus of these athletes should be to gain strength. These plyometric exercises may be miometric in nature to promote force absorption. ** Moderate-high intensity plyometric exercises may be prescribed as an effective rapid force production stimulus as athletes continue to gain strength. *** Moderate-high intensity plyometric exercises may be prescribed as the athlete has now gained sufficient strength to tolerate the overload stimulus provided by high intensity plyometric exercises.
